# KLOTHO‐VS heterozygosity ameliorates the effects of APOE ε4 on longitudinal hippocampal atrophy

**DOI:** 10.1002/alz.093899

**Published:** 2025-01-09

**Authors:** Linda Zhang, Eva Alfayate, Miguel Calero, Miguel Medina, Michel J. Grothe, Pascual Sanchez‐Juan, Bryan A. Strange

**Affiliations:** ^1^ Reina Sofia Alzheimer Centre, CIEN Foundation, ISCIII, Madrid Spain; ^2^ Network Centre for Biomedical Research in Neurodegenerative Diseases (CIBERNED), ISCIII, Madrid Spain; ^3^ Centre for Biomedical Technology, Universidad Politecnica de Madrid, Madrid Spain

## Abstract

**Background:**

KLOTHO‐VS heterozygosity (KL‐VShet+) has been posited to be a protective factor against age‐related disease and cognitive decline, having been associated with increased cortical volumes and brain connectivity, as well as improved cognition in healthy elderly individuals. Conversely, the APOE‐e4 allele is a primary risk factor for the development of Alzheimer’s disease (AD), with e4 carriers more likely to have greater ß‐amyloid burden, earlier age of AD onset, and accelerated rates of cognitive decline. Relatively few studies have investigated the interaction between these two genetic factors, with those that have presenting conflicting findings.

**Methods:**

720 cognitively normal elderly participants (mean age: 74.7 years, range: 69‐87) with 3T T1‐weighted MRI, APOE and KL‐VS genotyping were selected for cross‐sectional analyses from the Vallecas Project cohort, a single‐centre 12‐year longitudinal study with annual follow‐ups. Of these participants, 443 had at least two visits and were included in longitudinal analyses (average follow‐up 4.7 years). Linear regression models were conducted to study the interaction between KL‐VS heterozygosity (KL‐VShet‐ vs. KL‐VShet+: n = 539 and 181, respectively) and APOE‐e4 (carrier vs. non‐carrier: n = 135 and 585, respectively) on FreeSurfer‐derived hippocampal volumes, neuropsychological test scores, and plasma AD biomarkers (available for a subset, n=186), controlling for age, sex, education (in models with neuropsychological outcomes), and total intracranial volume (when considering hippocampal volume). Linear mixed‐effects models were used for longitudinal analyses of the same variables.

**Results:**

No significant interactions between genotypes were found for any cross‐sectional comparisons. A significant KL‐VShet+ by APOE‐e4 interaction was observed in longitudinal right hippocampal volumes, where KL‐VShet+ e4‐carriers had similar rates of hippocampal atrophy compared to e4‐noncarriers, and slower rates compared to KL‐VShet‐ e4‐carriers (ß=52.94, SE=25.03, p=0.03). No significant interactions were found for neuropsychological scores or biomarker levels.

**Conclusion:**

Our study provides additional evidence to suggest that KL‐VS heterozygosity attenuates the detrimental effects of APOE‐e4 on brain structure in healthy elderly adults, although the effects are not apparent in cognitive performance. Plasma AD biomarker levels did not vary significantly as a function of genotype, although this may be due to the smaller sample size. Further analyses will be conducted as more samples continue to be processed.

